# Sharing Visual Narratives of Diabetes on Social Media and Its Effects on Mental Health

**DOI:** 10.3390/healthcare10091748

**Published:** 2022-09-12

**Authors:** Syed Ali Hussain

**Affiliations:** Walter Cronkite School of Journalism and Mass Communication, Arizona State University, Phoenix, AZ 85004, USA; sahussa8@asu.edu

**Keywords:** visual narratives, social media, diabetes, mental health, chronic illnesses, Tumblr

## Abstract

Diabetes is a chronic illness affecting over six percent of the global population. Visual social media sites such as Tumblr provide a unique opportunity to understand visual illness narratives of type 1 and type 2 diabetes and its effects on mental health. We qualitatively analyze 259 Tumblr images with a “diabetes” hashtag. The results provide rich insights into the lives of diabetes patients, including personal and social life interactions, visual narratives portraying one’s acceptance and maintenance of diabetes, difficulty in social interactions, and how patients’ identity and beliefs are shaped by the daily struggles and failures of living with diabetes. We discuss the findings in the context of the chaos, quest, and restitution narratives of illness proposed by Arthur Frank. The results have implications for the visual representation of chronic diseases on social media and for improving patient–provider interactions and treatment of type 1 and type 2 diabetes patients.

## 1. Introduction

Diabetes is a chronic illness [1] and affects over six percent of the world’s population [2] Similar to other chronic illnesses, living with diabetes is a daily battle of self-care and decision making [3,4], patient education and psychosocial support, anxiety stress management [5], and better treatment adherence [6].

Diabetes patients have to follow a strict regimen in terms of diet and exercise. The regimens often bring communication breakdown between patients vs. caregivers, often referred to as “diabetes police” [7], or significant others (e.g., wife of a patient) overtly monitoring food consumption of diabetes patients. In such situations, many patients switch to social media for social support [8]. Social media enables them to share new forms of narratives that are quick, symbolic, and ad hoc [9], providing instant means for self-expression and identity formation [10]. In particular, visual narratives, such as photographs, help them to express in forms meanings that are otherwise not easily conveyed through words. Such visual forms of self-expression produce illness narratives that help patients with diabetes to make sense of life events, such as career, family, marriage, sex, and intimacy, among others.

Sharing narratives of personal experiences through social media is found useful in improved self-management behavior [11]. Illness narratives seem to have therapeutic effects for the writer and strongly influence the readers to understand the writer’s context [12]. Diaries, blogs, and other illness narrative expressions have also shown to produce therapeutic effects on social relationships and self-help [13].

Specifically, this study examines the visual illness narratives of diabetes on the Tumblr social media site. The findings have implications for employing visual narratives to improve chronic illness care for diabetes patients. Studying visual illness narratives can also help healthcare providers, researchers, and caregivers to understand the challenges that patients experience and can have design implications for better disease treatment.

### 1.1. Visual Research Methodology

Visual research methods have a long history in qualitative health research. For example, Kaley, Hatten, and Milligan [14] conducted a visual ethnographic study to explore how individuals with intellectual disabilities engaged in green care. Harvey and Brooks [15] explored the role of stock images in forming perceptions of patients with dementia. Cheezum, Rosse, Niewolak, and Cobb [16] conducted a PhotoVoice study to assess the determinants of health and wellness in Detroit’s homeless community. Additionally, researchers have used photo-videography in data collection and in the analysis of daily computing environments [17] and visual methods to explore human–computer interactions [18], including design projects and visual ethnography [19]. Other visual research methodologies include visual ethnography [20], ethnomethodology [21], documentary photography [22], photo elicitation [20], and photovoice [23]. Considering the abundant use of visuals on social media, visual research methodologies are also experiencing a transition from traditional objectivist forms of data collection and analysis to more subjective ways of looking into culture and social life as evidenced by more recent work in sociology and anthropology using photographs, illustrations, and videos [24,25].

### 1.2. Illness Narratives

Studies of illness narratives describe two distinct languages of discourse around health. The first is objective language, which includes medical information provided by physicians and other health providers. The second is subjective language, which talks about the intimate, non-verifiable experiences around having an illness. Frank [26] discussed the critical role of such subjective language in illness narratives as “ill people learn by hearing themselves tell their stories, absorbing others’ reactions and experiencing their stories being shared.” Illness narrative is shaped by a mix of interactions a patient has with other patients similar to himself, family, and providers. These could be in-person interactions and interactions taking place in mediated environments, such as websites or mass media (e.g., radio, movies, TV).

Researchers found that giving the opportunity to tell personal stories about their illness helps the participants to actively participate in their health improvement process [27]. In addition, by hearing stories from similar others they do not feel alone in experiencing the illness [26]. The conventional method to achieve this is through maintaining patient diaries. For instance, patients have produced narratives of their illness experiences and hospital stays in the ICU [28]. Healthcare providers have also recognized the importance of illness narratives. They use illness narratives to learn about the patient’s life after disease [29]. Intensive-care nurses use diaries to record their experience, which is often co-authored by other healthcare providers and even patients’ family members [30]. For example, a study examined the effects of digital photo-sharing on the social connectedness of patients with spinal cord injury and elderly living in a nursing home [31]. The results showed that the sharing of photos served as “food for the talk” and improved the bonding with family members.

### 1.3. Visual Narratives

With the presence of new media and communication technologies, people have found more elaborate ways to express their daily life stories. Among these, people use photographs to communicate through signs and symbols about concepts that are generally hard to convey in words [27]. The method has the potential to reveal hidden meanings as well as new opportunities to understand and improve disease management. Patients’ lived experiences might not always be possible to verbally describe. Narratives presented visually provide a unique window into the subjective experiences of the narrator [27]. For instance, a picture of swollen feet and the person’s painful gesture provides a window to see beyond physical symptoms and feel the emotional side of the patient’s struggle with the illness. Such visuals also show subtle details that are often not shared during medical visits. Visual narratives take several forms, including but not limited to photography [32] and graffiti [33].

The ubiquity of visual social media sites has led to a significant increase in the visual sharing of illness narratives, thereby increasing social interactions around it. This has been studied in many health contexts such as HIV AIDS [23], chemotherapy [19], and post-hospital recovery [32]. Visual narratives have also been used to study embodiment [34], body image, and how it shapes the personal–social identity [35]. To that extent, images provide a unique avenue to express and understand illness narratives with more richness.

### 1.4. Tumblr: A Place for Sharing Visual Narratives

**Figure 1:**
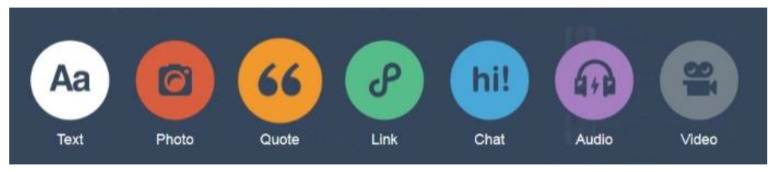
Tumblr dashboard.

Tumblr is a visual social media site that is popular among internet users, with its fast sign-up procedure and micro-text, images, and photographs sharing abilities. The website hosts over 518 million blogs [36]. It publishes over 12 million blog posts daily and has over 327 million unique visitors around the globe. The posts are categorized as photo, video, audio, text, link, chat, and answer (Figure 1). Several of these posts are re-blogs, with roughly 10% original posts [37]. In terms of posts’ sentiment, image posts express more intense emotions and positive valence compared with text-only posts [38].

Even though Tumblr appears similar to other visual social media sites, some differences still exist. For example, Pinterest lets users curate images. Flickr lets people view and like others’ photos but not share or re-blog. On Flickr, tagging behavior occurs significantly among friends. Accordingly, popular photos do not spread widely and quickly on the network. Over 50% of information exchange is between online friends but with a significant delay at each connection [39]. However, Tumblr makes possible the quick dissemination of information through the re-blogging feature, similar to Twitter.

Several researchers have used Tumblr blogs to study health topics. These include visual representation of anorexia and found blogs containing emotionally intense and triggering images [40]. Tumblr has also been used to examine visuals of domestic violence through a feminist participatory action approach [41].

Most research on illness narrative is based on textual data generated from written diaries, surveys, focus discussion groups, and in-depth interviews. However, qualitative data exist in other non-textual forms, such as signs, symbols, non-verbal gestures, paintings, and other visual artifacts. Visual communication offers significant opportunities to explore lived experience and the subjectivity of patients’ illness experience [27]. This is particularly relevant for patients with diabetes that puts them in awkward social situations such as having to inject a needle before a dinner date and having to worry about telling a blind date that one has a disease that is not curable. As patients tell their story, they untangle their illness experience and begin to accommodate to life as the person they have become.

Based on this discussion, this article presents a qualitative analysis of diabetes-related images on Tumblr and explores how patients use Tumblr in the context of expressing their illness. Our study informs how visual social media sites can be developed to effectively enhance patients’ illness experiences and help stakeholders such as providers and caregivers to better understand the patient’s perspective, leading to improved patient care.

## 2. Methods

### 2.1. Data Collection

First, the author searched the term “Diabetes” on Tumblr.com, which resulted in posts associated with the tag “diabetes”. Tumblr shows 15 posts on the first page and shows more posts as users scroll down. The search was repeated multiple times for four months. Each time new images were found. Those images were recorded in a dataset. Because we were interested in understanding how patients expressed their illness experiences in visual narratives, we collected only those posts that contained images. If the post contained images showing only text embedded in an image file (e.g., banner), the image was still included as our data. We captured the images and the text for each post. Posts that only included text or videos, but not images, were not captured. As a result, we collected 295 posts comprising photos, illustrations, cartoons, drawings, and images with embedded text. All posts were de-identified during the collection. We did not record the poster information and other meta-data, including re-blog and like counts, due to the scope of this study being exploratory. We focused our data collection and analysis on the images as units of analysis.

### 2.2. Data Analysis

We analyzed the images from Tumblr using open coding analysis [42]. We chose this method to identify emerging themes and patterns of an unknown phenomenon. The codes were iteratively refined and grouped using an affinity diagram [43] to make linkages among themes, categories, and sub-categories. The method allowed us to understand the social construction of illness experiences expressed by the posters. Additionally, the analysis provided an alternative understanding of patients’ beliefs and actions that may vary from those expressed in a clinical setting [43]. We asked the following questions during the analysis:

1. What is portrayed in these images?
2. What diabetes-related problems are highlighted in the images?
3. How does the poster cope with the problem, if any?
4. What are the distinctive characteristics across the images?
5. What constrains or facilitates the process of disease management?

With these questions in mind, the author began the descriptive coding, translating the visual images into a textual description of what each image contained using Atlas.Ti qualitative software. The textual data were also coded using open coding schema. Next, the first author moved on to interpretive coding, identifying possible causes, implications, and connections among the images by iteratively comparing images and codes produced from the descriptive codes. The author then conducted axial coding, attempting to find emerging themes of clusters and connections among the codes.

For eight months, the author regularly discussed and debated the concepts and themes arising from data with a team of researchers once a month at research meetings. The meetings comprised graduate students and a faculty member with expertise in health and risk communication, communicative science and disorders, games, and human–computer interactions. The author solicited feedback from the group on the interpretation of the images, gaining consensus on balancing interpretation with a more objective view of what each image conveyed relative to the poster’s illness experiences. Comments and questions generated from this process provided further avenues for reflection during the analysis. The quotes from the textual images appear in italics with double quotation marks throughout the article.

## 3. Results

### 3.1. Finding 1: The Beginning

Tumblr users posted metaphors, body images, illustrations, cartoons, selfies, photoshopped images, memes, and photographs of their daily experiences. These images expressed their frustration of accepting diabetes as part of their lives, dealing with fluctuating emotional and physical challenges, along with the determination to manage their illness.

### 3.2. Acceptance of Diabetes as a Chronic Illness

Tumblr users posted revelations about how long it took to embrace both the good and the worse parts of living with diabetes. Seeing other Tumblr users go through similar situations helped them to move on to accepting the disease. One post stated: *“It is never really been easy to talk about [diabetes] to other people, but I have come to realize over time that diabetes has become a big part of me. It’s been the greatest part (I literally get to eat skittles … daily), and the worst part (I … get to take insulin shots … daily). But it’s something that I can’t change.”* Here, the quote illustrates the poster’s coming to agreement with “the greatest” and “the worst” parts about having diabetes as a major life transformation.

**Figure 2:**
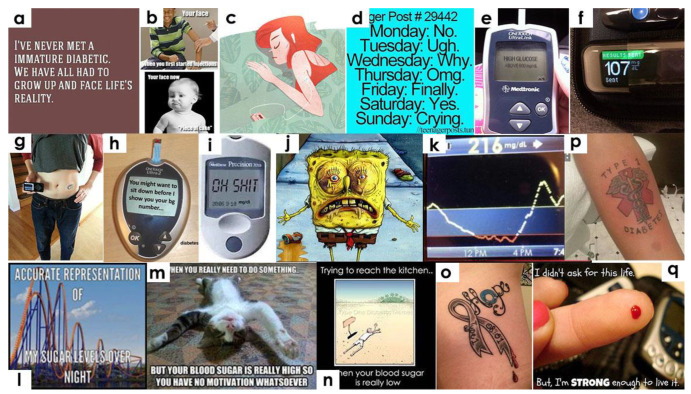
Images related to the beginning of diabetes and related illness narratives on Tumblr.

Another poster, after spending 15 years with diabetes, accepted “the fact that I’m a diabetic” and stated how “Finding this blog has helped so much, and knowing others struggle the same as I do [was] reassuring.” Similarly, another poster stated: “I have never met an immature diabetic. We have all had to grow up and face life’s reality.” (Figure 2a). One poster talked about learning from the past, when injecting insulin was a big deal, which has now become a “peace of cake.” (Figure 2b). The images of these posts showed peoples’ perceptions about the self and the illness transforming overtime, finally leading to a lifelong commitment to the illness.

### 3.3. Emotional and Physical Fluctuations

Tumblr images depicted the emotional fluctuations and physical symptoms that people with diabetes pass through on a daily basis. For example, one post expressed how people with diabetes find it hard to have a good peaceful sleep, showing an image of a woman sleeping peacefully (Figure 2c). Another post, tagged as “anger post,” described their weekly routine as: “*no, ugh, why, omg, finally, yes and ends at crying*” (Figure 2d), showing the constant challenges they face with diabetes throughout the week. Another poster expressed: “*I hate my life and need to roll over and die”* (Figure 2e). Another image showed that diabetes “*loves to complicate*” things in their life. Other posts showed similar feelings of frustration when they have important work to do or meet deadlines. These images showed the emotional trauma and despair that diabetes patients pass through daily as they struggle to manage blood glucose.

Measuring blood sugar takes a large part of diabetes patients’ everyday life. This was evident through the substantial number of images about glucometers and insulin pumps in our data (Figure 2f). Posters expressed several emotions surrounding the use of a glucometer, for example, anxiously waiting for the glucometer and the excitement upon finally receiving it. Reading the instructions and using the glucometer for the first time held particular importance (Figure 2g). In addition, images conveyed the emotional state of waiting for the blood sugar number to appear on the glucometer and expressed feelings of extreme disappointment upon receiving a high blood sugar number (Figure 2h). For instance, one image showed a glucometer screen displaying “*Oh Shit*!” instead of the actual glucometer reading (Figure 2i). The images showed their constant fear of uncontrolled diabetes. This image was related to another image showing the cartoon character Sponge Bob Square Pants (Figure 2j) depicting panic attacks and depression. The findings consist of a mix of both excitement and despair, depending upon the test results. We realized that it is not only the physical feeling but also the emotional state of sadness upon receiving a low glucometer reading that adds up to their stress.

Feeling low (blood sugar) or high (blood sugar) are critical indicators for tracking how well blood sugar is controlled. Feeling low happens when patients’ medications are overworked compared with the level of sugar in their blood. Patients need to take sugar as a remedy not to fall into shock. Diabetes patients can feel low at one point and high the next. Many posts expressed such fluctuating physical symptoms as contributing to fluctuating emotional states. For instance, a screenshot image of a fluctuating blood sugar monitor (Figure 2k) was coupled with the text: “*a long day.”* Another image expressed the low sugar levels at night as similar to a frantic roller coaster (Figure 2l). When patients are low, they want to keep sleeping, sedated with sugar, tired of the struggle, and scared of the consequences (Figure 2m). Some posts described the state of feeling low and attempting to take sugar as crawling on a desert: *“Trying to reach the kitchen…when your blood sugar is very low”* (Figure 2n). We found strong indications of emotional burnout and fatigue in managing the illness throughout the day. Additionally, there is no particular time of day when they could be feeling worse or better. Instead, images related to feeling high and low appeared across the board at all times of the day.

### 3.4. Determination to Fight against Diabetes

Tattoos emerged as a dominant theme in our data (Figure 2o), mainly as an expression of determination and hope. These images included a tattooed text, shape, or design that captured their health condition. The designs expressed emotions such as hope, the cycle of life, dependency on disease, and expressions of staying calm while bravely surviving the illness. One post read *“Tatted up finally. Type 2 Diabetes for five years now.”* (Figure 2p) Despite all difficulties, posts shared encouraging messages and showed determination to keep fighting with the disease until its cure is found (Figure 2q).

Posts other than tattoo images also conveyed determination. For instance, a post showed comic hero characters such as “insu-man” and captain glucose to keep them driven. Many posts compared improvements made over time and the associated pride with these accomplishments.

In sum, the images portrayed their beginning journey as a diabetes patient, starting with struggles and acceptance, while continuously being challenged with emotional and physical fluctuations, but being determined to stay healthy. Although posters often used symbolic metaphors that only insiders would understand, the posters attempted to talk to the broader audience about how challenging it is to go through this initial phase of having diabetes.

### 3.5. Finding 2: Living with Diabetes

**Figure 3:**
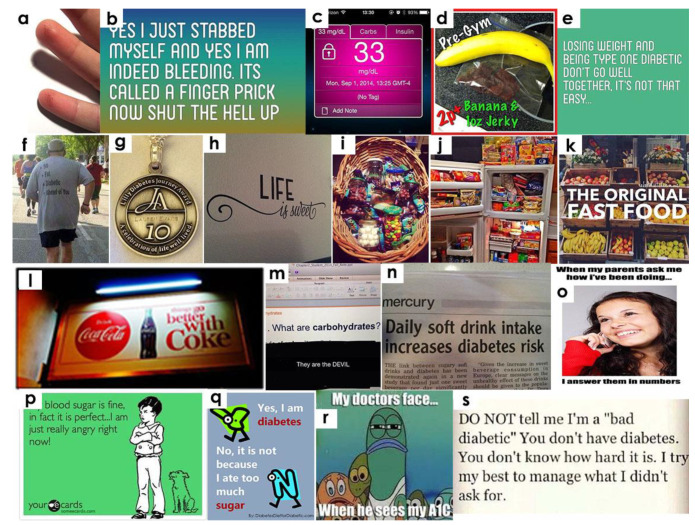
Images related to the maintenance of diabetes posted by Tumblr users.

Posters also expressed the need for others to understand the daily struggles of a person with diabetes. We found several posts indicating that people with diabetes strongly wish others could feel their emotional and physical pain. Many images were shared about the misunderstandings and stereotypes associated with diabetes. One post read: *“Don’t tell me I am a bad diabetic. You don’t have diabetes. You don’t know how hard it is. I try my best to manage what I didn’t ask for”* (Figure 3s).

Several other posts expressed a similar level of annoyance regarding when someone says too much sugar relates to diabetes or when people mix up type 1 with type 2 diabetes. Many posters tried to raise awareness regarding type 1 diabetes, which is not limited to children only and it is not always caused by eating sugar: “*I have type-1 diabetes because I ate too much sugar*” (Figure 3q). A similar post showing what people with diabetes do not agree with stated: “*you didn’t care for yourself*.” On the whole, we found posters trying to clarify that there are no good or bad diabetes patients and that each patient is different in terms of acquiring and experiencing the illness.

Speaking about the daily social interactions, one post mentioned: “*people with diabetes notice the knee-jerk responses by others when they order low-calorie food in a public space*.” Another post mentioned that people with diabetes feel bad and hurt when they are asked to check their blood sugar and strongly want other people to stop judging them. Overall, we found that people with diabetes react with hostility towards social control and pressurizing behavior of others.

With regards to diabetes, information spread through mass media was found to be both helping and misleading. For example, posters mentioned that mass media spreads the misperception that blood sugar is correlated with anger. One post mentioned a person with diabetes hearing from others: *“You are not fat, you don’t look fat.”* Few posts touched upon the misperceptions that exist within the diabetes community, such as the proper usage of flex pens vs. syringe and the right way to inject insulin. Mass media have also helped raise awareness about diabetes; for example, one poster thanked news reporters for clarifying that: “*daily soft drink intake increases diabetes risk*” (Figure 3n). On the whole, we found a considerable portion of images posted on Tumblr about mass-media-based messages on diabetes. We noticed that Tumblr users aggressively responded to such misperceptions and showed a strong urge to clarify these misperceptions as much as they could.

### 3.6. Interactions with Friends, Family, and Providers

Tumblr images also included different ways a person with diabetes interacts with family, friends, and healthcare providers. These posts revealed the close monitoring of their parents on the posters’ diabetes status, which the posters took as sometimes excessive and sometimes supported. For example, one post mentioned: “*When parents ask, ‘how you are doing?’ I reply in numbers. My friends don’t understand what I said, but my parents do.”* (Figure 3o). Another image showed parents with an insulin pump painted on their stomach as a gesture of support to their child who has diabetes. Other posts showed images of adolescent diabetes patients being panicked upon seeing high glucose readings but trying to hide this from parents. A similar post stated: “*Sometimes when I act stupid, my blood sugar is just fine…so don’t ask me to check it.”* Another post read: “*My blood sugar is fine, in fact, it is perfect…I am just really angry right now!”* (Figure 3p). All these images expressed frustration and distress about the comments they receive from others who do not know about diabetes. We found that posters strongly protested about being judged by others for having diabetes or not managing their illness.

Tumblr users portrayed their friends as being unsupportive or uninformed about their diabetes status. One image showed how posters illustrated their friends being the diabetes police—meaning that the posters felt that the friends judged the posters’ behavior. One quote mentioned above illustrated that friends did not understand the diabetes conversation they have with their parents. We also found images in which posters strongly opposed the misperception that a person gets diabetes because their parents did not take care of them (Figure 3q). Few posters showed their gratitude towards parents and appreciated their help, albeit over-protective, in correcting their diet and exercise.

We also found posts related to interactions with healthcare providers. For example, some posts mentioned to avoid self-diagnosis and consult a doctor if you think you have diabetes. One image mainly talked about the perceived facial expressions of the doctor when he knows the A1C score (Figure 3r). Overall, we found a tendency of hiding diabetes-related worries from both friends and family. At the same time, we noticed a silent want of support from diabetes patients. They perceived their larger social circle as being unsupportive and ignorant to their real condition. Overall, we found reluctance towards both friends and family members as the groups to open up to about their situation.

### 3.7. Finding 3: Self-Reflection

This section describes findings around Tumblr images portraying the posters’ reflection about their illness. This section describes the core value that illness narratives offer—the beliefs that patients have and how those beliefs drive their behavior; rich, historical stories of failure, recovery, and restoration; and how those experiences built up who the patients are.

### 3.8. Life: Past, Present, and Future

**Figure 4:**
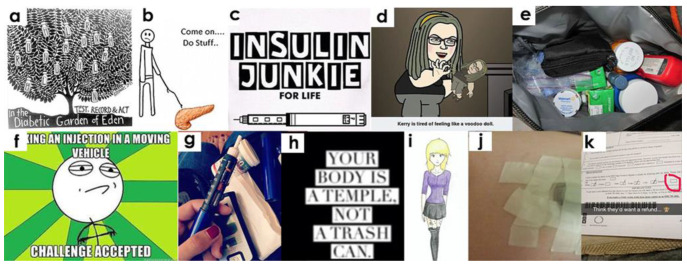
Images related to the self-reflection on diabetes.

Our analysis of Tumblr images found that people with diabetes expressed a profound view of both past and future about the illness. In terms of looking to the past, we found images about how their health had improved or worsened with time. In many images, patients expressed that they hate to think about past events that they do not want to remember. We also found forward-looking images in which people with diabetes expressed a mix of hope and distress. One such post mentioned that distress is associated with how many more years one would have to live with diabetes, especially in the absence of a permanent cure. Another image showed the Garden of Eden in heaven with a tree’s hanging fruits being insulin bottles (Figure 4a). Overall, we found a mix of bittersweet experience with regards to diabetes management. It is not surprising considering the chronic nature of the disease; however; we think that feelings differ depending on the time spent with the illness. Although we could not assess the age of the person posting on Tumblr, we found indications that older patients seemed to have negotiated with their illness as they made an effort to maintain health and wait for better cures.

We also found a strong sense of helplessness expressed in the images posted on Tumblr, such as expressing feelings of depression because of the restrictions imposed by the illness (Figure 4b). One such image showed that diabetes had defined their territory, which is limited to the glucose test result. Explicitly, the younger patients expressed added frustration of not being able to enjoy “*junk”* food, which they loved so much. One post expressed the same feeling of reliance and helplessness by relating diabetes with a voodoo doll controlled by strings in the hands of the disease (Figure 4d) and thinking of oneself as an insulin junkie for life (Figure 4c). Some posts indicated that people with diabetes find it hard to believe that their whole life depends on the medical supplies in a pouch that continuously dictates their daily choices and actions (Figure 4e). Some posts talked about the added complexity of diabetes when coupled with other illnesses such as the common cold. Overall, we found that living on the terms and conditions defined by diabetes is particularly frustrating for diabetes patients. We noticed that diabetes patients longed for the past when they had the freedom and liberty to enjoy food.

We also found images in which posters talked about accepting the disease as a permanent part of their life, again with an understanding of there being no escape from its complexities. They showed to console themselves by thinking that diabetes is a disease with a long lifespan, and they can survive it even with low-functioning organs. We found images in which people appreciated the invention of insulin but with the recognition that it is not a replacement for a fully functional pancreas. Images depicted the reality of being used to insulin shots, daily monitoring, and pricking with the realization that it is not getting any easier. With regards to reactions towards glucometer readings, we found posters expressing optimism to keep the morale high and hope for a better score in the coming months. On the whole, we found that people with diabetes are fully aware of the fact that their illness is without cure and that it may become worse if they do not put in concerted effort to control it.

With regards to diabetes management, we found images related to routine day-to-day activities and their impact on the illness. For example, one poster mentioned that working from home is particularly helpful for a person with diabetes. A post about medication intake mentioned that unlike other illnesses, diabetes patients do not have a fixed time for medication because it depends on their glucometer reading and daily fluctuations in the sugar level. One post was about the extra space needed to carry diabetes supplies such as medicines and the glucometer. Few posts talked about the challenge of having to inject insulin in a moving vehicle (Figure 4f). Another image showed insulin injection as an old, long-time friend (Figure 4g). With regards to educating newly diagnosed patients, one post explained the difference between CGM and an insulin pump by clarifying that CGM measures blood sugar levels and not insulin.

### 3.9. Self-Perceptions about Body Shape and Organs

The images posted on Tumblr also showed how people with diabetes might view their physical body and internal body organs, especially stomach and pancreas, in the context of the illness. One image showed that people with diabetes tend to treat their body as a very sacred place, such as a temple, and critically reject the views of treating it as trash where one can just throw anything (Figure 4h). Other posts showed that people with diabetes idealize a very smart and slim body (Figure 4i). One post talked about the color and size of the insulin pump because this determines its placement on the body and also the effect on the choice of clothing. In the case of the stomach, images showed insulin pumps pasted with tape (Figure 4j). One post showed a patient filling out a driver’s license form and not checking the field for organ donation of the pancreas (Figure 4k). Other posts showed a healthy body but a dead pancreas. Such posts expressed emotions associated with sadness because the pancreas does not perform as well as other body parts. Another image showed a cartoon character talking with his pancreas and saying: *“Diabetes, you will not compromise recovery!”* From these posts, we found that people with diabetes are very conscious of their physical changes and how the illness affects their perception about their physique and perceptions of smartness. It is not surprising that the pancreas and insulin production remain a dominant thought on the minds of diabetes patients. Thus, anything related to pancreas either on mass media, print advertisements, or news immediately catches their attention. Additionally, diabetes patients stay on the lookout for more ways to paste the insulin pump on their body. The stomach is the most used place for this because it hides the pump under the clothing.

## 4. Discussion

The ultimate goal of this study is to examine how visual illness narratives can be used to help various stakeholders, such as peer patients, health professionals, researchers, and caregivers, to better provide support for patients. We learned that micro-visual illness narratives provide a rich medium for Tumblr users to express struggles, share help, and reflect in depth. The images triggered productive conversations about posters’ illness experiences that we might have not otherwise captured elsewhere. Below, we discuss how visual narrative illness extracted from Tumblr images provide utility and implications around patient care.

### 4.1. Chaos, Quest, and Restitution Narratives of Diabetes

In his book *The wounded storyteller: Body, Illness, and Ethics*, Frank classified three broad categories of illness narratives, i.e., restitution, chaos, and quest narratives (Frank, 1995). Based on these classifications, we elaborate on the illness narratives found on Tumblr. First, *chaos narratives* are disjointed and without temporal sequence. Tumblr images showed posters’ progress in their diabetes management to fluctuate between sick vs. worse, no matter how hard they tried to maintain it. As a result, posters showed signs of despair and loss of hope. Chaos narratives describe experiencing illnesses with no cure and/or unreliable treatments. Patients suffering from chronic illness find listening to chaos narrative helpful because they no longer feel alone.

Patients with experiences associated with the narrative are often not in a position to fully articulate their condition. Accordingly, chaos narratives are difficult to retrieve from patients. Close family members and friends mostly witness such narratives. However, through Tumblr images, the public could witness chaos narratives around posters’ earlier struggles and acceptance of the illness. The narrative depicted anxiety, frustration, and distress from illness. It revealed vulnerability and weaknesses, which most people hide, thus helping us understand the more in-depth, subjective side of the illness experience.

*Quest narratives* are about patients’ fighting back. These illness narratives consist of stories about patients’ strong will and making a concerted effort to fight the disease. Accordingly, the narrative includes signs of improvement. Quest narratives also depict alternative ways of being well by focusing on how to live longer and healthier. We found quest narratives to be most commonly appearing after chaos narratives on Tumblr. Patients’ quest narratives consisted of images showing efforts to manage the diet, perform regular physical activity, and showing a positive attitude towards life, even though pricking emerged as one of the most painful aspects of patients’ life, even when posters showed signs of resilience and commitment to get better.

*Restitution narratives* point toward the belief that health is restorable. These narratives are stories of the recovery and restoration to better health. For example, “in the past, I was healthy, today I have an illness, but I will be healthy again tomorrow.” The restitution narrative originally denotes recovery. The connection between the restitution narrative and our data was the hopefulness and positive attitude of posters. For instance, images showed recovery and improvement in maintaining the glucose level, performing physical activity, controlling the diet, battling temptations to eat sugary food, and managing stress with a sense of optimism and hopefulness for the future self. These images provided insights about the everyday matters of people with diabetes and subtle realities that they may not openly disclose in a clinical setting. Tumblr also provides users with an opportunity to express aspects of their illness that may be considered taboo to openly share. Such narratives can open up conversations about sensitive topics that many want to talk about but never find the opportunity to do so.

### 4.2. Implications for Stakeholders and Chronically Ill Patients

Tumblr images with the diabetes hashtag showed rich expressions of diabetes illness narratives. The images communicated the anxiety, frustration, and vulnerability of posters who might have found it difficult or not appropriate to easily express such emotions in an offline social setting. We also found stories of hope and struggle in which posters were seen facing the illness with a strong will to get better. Such narratives consisted of posters who made a concerted effort to fight the disease and showed enormous signs of improvement. These images also showed alternatives ways of being well by focusing on how to live longer and healthier under the existing health conditions.

Social media are increasingly becoming mostly visual media, and enormous possibilities exist to utilize these data for pro-social benefits. Our analysis contributes unique insights about how diabetes patients self-disclose their illness narratives and experience on Tumblr. Patients and researchers can use the data to understand patients’ experience, follow one’s trajectory of illness, and emerging trends by predicting possible outcomes from a patients’ current physical and emotional state. For instance, we can use crowd-sourced tags, meta-data of images, and automated visual analytic technologies to group images showing struggles, success, tips, and self-reflection. These groups can be used to provide tailored help to peer patients going through struggles. Posters can be matched as recommended friends to provide peer help if they post about similar emotional distress status.

Researchers can use these images to understand emerging trends in a cross-cultural context. Currently, Tumblr posters are predominantly western-oriented. However, with time, Tumblr membership may grow to include other nationalities, thus providing even richer insights for diabetes management in an international public health setting. Research organizations such as the Center for Disease Control and the National Cancer Institute can benefit from these findings to formulate funding announcements employing visual illness narratives on social media to better understand and serve the diabetes community.

During the analysis, a strong sense of disapproval was found towards the social stereotypes and misunderstandings about diabetes in the Tumblr community. We suggest that this topic be brought into discussion by healthcare providers during hospital visits and counseling sessions to provide patients with an opportunity to express and build strong patient–provider relationships. In addition, diabetes support groups and community health educators can use this information for community engagement and for supporting existing health education interventions. For instance, patients with speaking disabilities or limited English proficiency may find it hard to verbalize their illness experience. Such patients can be shown visuals to use as a medium for expressing their concerns and questions.

Additionally, the findings of this study can contribute to the design and development of glucometers, insulin pumps, and future wearable devices for diabetes care. We found specific emotional and physical behaviors associated with the use of these mobile devices that have implications in patients’ choice of clothing and preference for mobile devices. For example, children with diabetes may show specific resistance to wearing an insulin pump. It is both uncomfortable and insecure for them to wear during school and play. The findings can help design future devices which are more convenient and more comfortable to wear for all age groups.

The study findings also have implications for health marketing and advertisement. We found that people with diabetes have a critical view of mass media advertisement, specifically about food products. A strong sense of skepticism and lack of trust was seen towards soda and sugary food manufacturers. Health marketers can use social media images to gain insights and design useful print and mass media advertisement and public service messages.

Our study is not without limitations. We do not know detailed information about the posters in terms of demographics and whether they were diagnosed patients. Our findings are instead based on the analysis of the images that the posters shared on Tumblr. Interviewing the Tumblr posters of these would help us to further understand how our interpretation differed and matched with the posters’ intention. Our next step is to involve healthcare providers, caregivers, and patients to extract their interpretations of these images. This process could unveil hidden communication breakdowns and hints for improving patient–provider relationships.

## Data Availability

The images used in the analysis are embedded within the manuscript. All data used in the study is publicly available on Tumblr.
